# Short-term effects of air pollutants on hospital admissions for asthma among older adults: a multi-city time series study in Southwest, China

**DOI:** 10.3389/fpubh.2024.1346914

**Published:** 2024-01-29

**Authors:** Yuqin Zhang, Xi Yang, Wanyanhan Jiang, Xi Gao, Biao Yang, Xing Lin Feng, Lian Yang

**Affiliations:** ^1^School of Public Health, Chengdu University of Traditional Chinese Medicine, Chengdu, China; ^2^School of Public Health, Peking University, Beijing, China

**Keywords:** air pollution, asthma in older adults, economic cost, generalized additive model, time-series study

## Abstract

**Background:**

This study aimed to explore the relationship between air pollution and hospital admissions for asthma in older adults, and to further assess the health and economic burden of asthma admissions attributable to air pollution.

**Methods:**

We collected information on asthma cases in people over 65 years of age from nine cities in Sichuan province, as well as air pollution and meteorological data. The relationship between short-term air pollutant exposure and daily asthma hospitalizations was analyzed using the generalized additive model (GAM), and stratified by gender, age, and season. In addition, we assessed the economic burden of hospitalization for air pollution-related asthma in older adults using the cost of disease approach.

**Results:**

The single pollutant model showed that every 1 mg/m^3^ increase in CO was linked with an increase in daily hospitalizations for older adults with asthma, with relative risk values of 1.327 (95% CI: 1.116–1.577) at lag7. Each 10 μg/m^3^ increase in NO_2_, O_3_, PM_10_, PM_2.5_ and SO_2_, on asthma hospitalization, with relative risk values of 1.044 (95% CI: 1.011–1.078), 1.018 (95% CI: 1.002–1.034), 1.013 (95% CI: 1.004–1.022), 1.015 (95% CI: 1.003–1.028) and 1.13 (95% CI: 1.041–1.227), respectively. Stratified analysis shows that stronger associations between air pollution and asthma HAs among older adult in females, those aged 65–69 years, and in the warm season, although all of the differences between subgroups did not reach statistical significance. During the study period, the number of asthma hospitalizations attributable to PM_2.5_, PM_10_, and NO_2_ pollution was 764, 581 and 95, respectively, which resulted in a total economic cost of 6.222 million CNY, 4.73 million CNY and 0.776 million CNY, respectively.

**Conclusion:**

This study suggests that short-term exposure to air pollutants is positively associated with an increase in numbers of asthma of people over 65 years of age in Sichuan province, and short-term exposure to excessive PM and NO_2_ brings health and economic burden to individuals and society.

## 1 Introduction

Bronchial asthma (abbreviated as asthma) is a common chronic respiratory disease with clinical manifestations of wheezing, shortness of breath, chest tightness, and cough (1, 2). So far, about 330 million people worldwide are suffering from asthma, which causes a heavy medical burden (3). Meanwhile, the pace of population ageing is much faster than the past, and the number of older patients with asthma is expected to increase significantly (4). The prevalence of asthma in people over 65 years of age is currently reported to be 4%–13% globally (5), and this is likely to be an underestimate (6). Asthma surveillance data released by the US Centers for Disease Control and Prevention (CDC) showed that the asthma prevalence among those aged ≥65 years in the US in 2020 was 7.8% (7). Notably, the 2010 to 2012 China Asthma and Risk factors Epidemiologic survey (CARE) reported that the prevalence of asthma in China was 2.26% in people aged 61–70 years and 3.10% in those aged ≥71 years (8). while the data from 2012 to 2015 China Pulmonary Health (CPH) study showed that asthma prevalence increased to 6.0% in people aged 60–69 years and to 7.4% in people aged ≥70 years (9).

Asthma results from the combined action of genetics and environment, with air pollution being an important factor in triggering asthma (10) and its exacerbation (11, 12). Even when the pollutant concentrations are lower than guideline levels, there are still have serious effects on susceptible individuals (13). Several mechanisms reasonably explain how air pollutants contribute to the development and exacerbation of asthma, including eosinophilic and neutrophilic inflammation through stimulation of airway epithelium, increased production of pro-inflammatory cytokines, oxidative stress, and deoxyribonucleic acid (DNA) methylation changes (14, 15), these changes subsequently induce sensitization to airborne allergens and allergic inflammation (14). With the increase of age, lung function appears physiological decline (16). Compared with young patients with asthma, older adults patients with asthma show more severe symptoms and have an increased risk of frequent medical visits and even death (17), which indicates that they are more likely to consume medical resources due to air pollution (18) and have a greater economic burden (19).

Asthma in children and adults has long been a concern (20, 21). However, numerous studies have shown that the prevalence of asthma increases with age (8, 22, 23). So far, there is limited evidence that air pollution increases the risk of medical visits in older adults with asthma (24, 25). Studies in China have indicated that air pollutants have adverse effects on asthma in older adults. A study in Beijing (26) found that for every 10 μg/m^3^ increase in PM_2.5_, hospitalizations for asthma increased by 0.67%. In contrast, Zhang et al. (27) analyzed daily records of respiratory disease hospitalizations and air pollution data in Shenzhen during 2015–2016 and did not identify an association between asthma hospitalization and PM_10_ or PM_2.5_. Analysis by Luo et al. (18) found that every 10 μg/m^3^ increase in SO_2_ was associated with a corresponding 7.27% increase in hospitalizations among older adults patients with asthma; whereas PM_2.5_, PM_10_, and NO_2_ were not associated with asthma exacerbation. Noticeably, these studies were primarily conducted in economically developed areas (20, 28), and most of them were single-city studies. Sichuan has become one of the regions with the worst combined air pollution in China (29), and there are large differences in concentrations of different pollutants temperature and humidity in different cities (30). However, current researches mainly focused on the provincial capital city Chengdu (31, 32), with studies in multiple cities in this province lacking.

To fill this data gap, in this study, we aimed to assess the associations between short-term exposure to air pollutants and hospital admissions (HA) of asthma in older adults people over 65 years of age, and quantify the corresponding health and economic loses of asthma caused by PM and NO_2_ pollution, with these two hypothesis: (1) short-term exposure to air pollution is positively associated with acute exacerbation of asthma in older adults patients in Sichuan province resulting in being hospitalized; (2) excess air pollutants may increase the economic burden of asthma-related diseases among older adults.

## 2 Materials and methods

### 2.1 Study area

Sichuan province is located in the southwestern region of China and consists of two major parts, the Sichuan Basin in the east and the western Sichuan Plateau. Sichuan province is ranked as one of the most heavily polluted areas in China due to high pollutant emissions and its unique topography which is not conducive to atmospheric dispersion (33). Meanwhile, Sichuan has a large older adults population with 14.168 million people aged ≥65 years, ranking second in China following Shandong (34).

**Figure 1:**
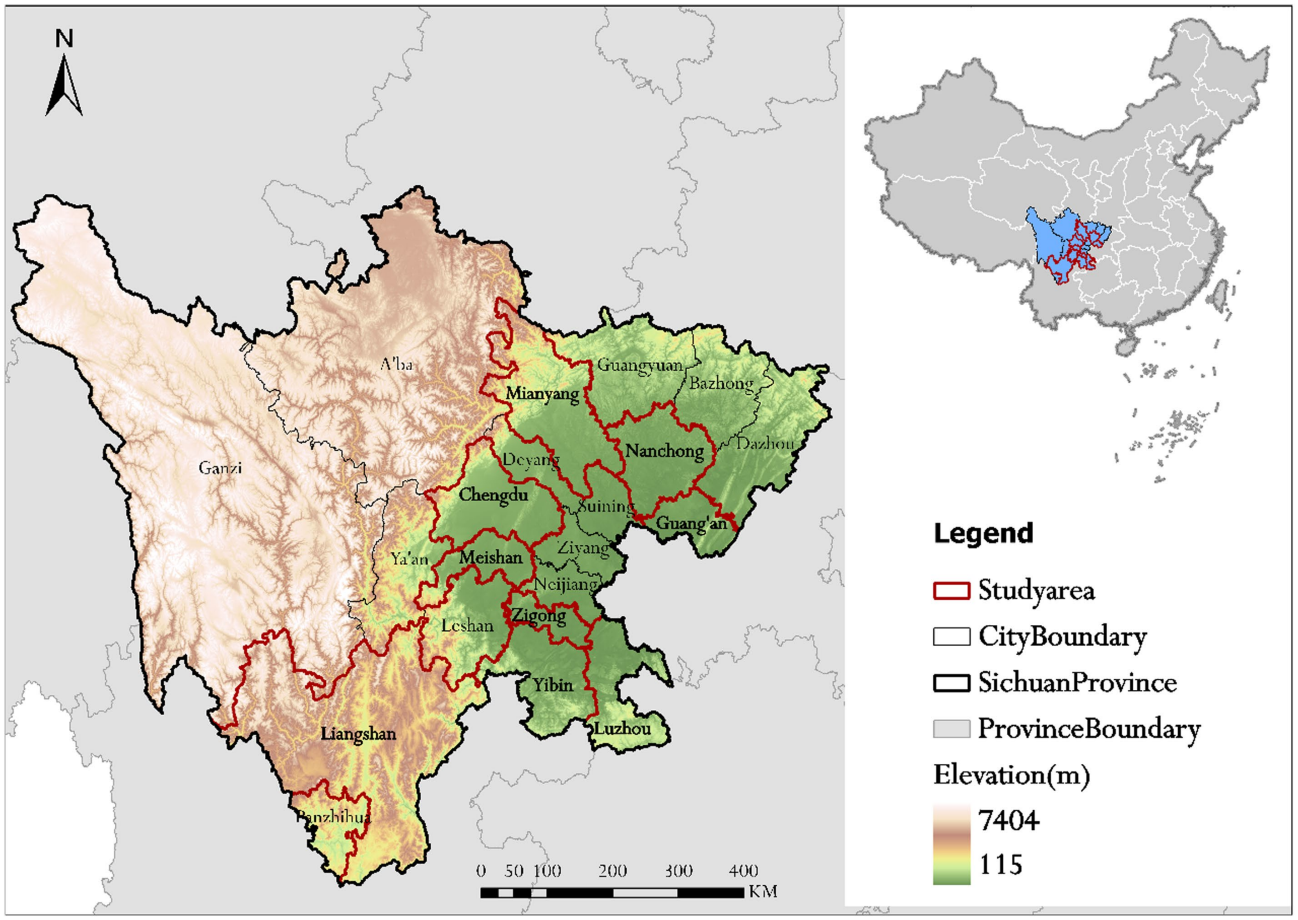
Geographical distribution of the study areas, 9 cities in Sichuan province.

Sichuan province has a total of 21 cities/prefectures, including 18 cities in the Sichuan Basin and 3 autonomous prefectures in the West Sichuan Plateau. Medical institutions in nine cities and prefectures across Sichuan province, including Chengdu, Mianyang, Nanchong, Guang’an, Meishan, Zigong, Yibin, Luzhou and Liangshan Yi Autonomous Prefecture, were selected for the study, of which 8 were from the Sichuan Basin and 1 from the West Sichuan Plateau, and medical institutions are all types of hospitals in the area, including general hospitals, Chinese medicine hospitals, specialist hospitals and private hospitals, representing the overall situation of Sichuan province to a certain extent covering different economy and population (Figure 1).

### 2.2 Data sources

Information on asthma patients aged ≥65 years between January 1, 2017 to December 31, 2019 was collected from hospital electronic health records (EHRs) of 273 hospitals in 9 cities of Sichuan province, with data variables including gender, age, home address, date of birth, hospitalization and discharge dates, and total cost of hospitalization. The International Classification of Diseases tenth edition (ICD-10) codes, including J45 (asthma) and J46 (status asthmaticus) were used to identify asthma-related hospitalization cases. Additionally, hospitalization data for 6,092 asthma patients aged ≥65 years were identified. This study was approved by the Ethics Committee of Hospital of Chengdu University of Traditional Chinese Medicine (Approval No. 2020KL-001).

Daily average levels of CO, NO_2_, SO_2_, O_3_, PM_10_, and PM_2.5_ during the study period were obtained from the Sichuan Environmental Monitoring Center. During this period there were 82 air monitoring stations in the 9 cities and prefectures: 19 in Chengdu, 9 in Mianyang, 9 in Nanchong, 6 in Guang’an, 6 in Meishan, 6 in Zigong, 17 in Liangshan Prefecture, and 10 in Yibin. Meanwhile, meteorological data, including daily average temperature, daily average relative humidity, atmospheric pressure, and wind speed were obtained from Sichuan Meteorological Bureau.^1^

We adopted inverse distance weighting (IDW) method to estimate the exposure levels of the individual cases (35). Specifically, the locations of all monitoring stations and home addresses of hospitalized asthma patients were geocoded using the Gaudet Map API,^2^ the next, for each older asthma patient, the inverse distance (1/*d*^2^) weighted average of the concentrations from all monitoring stations was used to assess the air pollutant exposure for a specific period of hospitalization, including single-day lag exposure (lag0–lag7) and multi-day moving average lag exposure (lag01–lag07).

### 2.3 Study design and statistical analyses

#### 2.3.1 Effects of air pollutants on asthma HAs

The collected patient information, pollutants and meteorological data were statistically described. Spearman’s rank correlation coefficient was used to analyze the correlation between air pollutants and meteorological factors, with absolute values of the correlation coefficient *r* closer to 1 indicating a stronger correlation.

The relationships between asthma hospitalization and explanatory variables are mostly nonlinear (20). Therefore, we use generalized additive model (GAM) to evaluate the association between ambient air pollutants and hospitalizations of older adults patients with asthma. Amongst the entire population, hospitalization for asthma is a low-probability event, and its actual distribution approximates a Poisson distribution. Therefore, a GAM with quasi-Poisson regression was used to analysis the associations between the six air pollutants (CO, NO_2_, PM_10_, PM_2.5_, SO_2_, and O_3_) and the daily asthma hospitalizations, while controlling for the effects of confounding factors such as long-term and seasonal trends, meteorological factors, and day-of-week effects. Previous studies have shown that the lagged effect of air pollutants were usually short (36, 37). In this study, single-day lags (from lag0 to lag7) combined with multiple-day lags (from lag01 to lag07) were applied to evaluated the lagged effects of air pollutants. The model is as follows Equation (1):


(1) $$\begin{array}{c} \log\left[E\left(Y_{t}\right)\right]=\alpha +\beta Z_{t}+s\left(time,k=df+1\right)+s\left(Temp_{t},k=df+1\right)\\ +s\left(Rh_{t},k=df+1\right)+s\left(P_{t},k=df+1\right)\\ +s\left(W_{t},k=df+1\right)+DOW+Holiday \end{array}$$

where $Y_{t}$ is the number of asthma hospitalizations on day *t*; $E\left(Y_{t}\right)$ is the expected medical visits for asthma on day *t*; *β* is the regression coefficient; $Z_{t}$ is the pollutant concentration at a certain lag day, (unit μg/m^3^or mg/m^3^); s is the smooth function; k is the degrees of freedom df+1 ; DOW is the day of the week; Holiday is a binary variable for national holidays in China; time is the date, and the degrees of freedom in the model are taken according to the literature and the principle of minimum AIC value in the Akaike information criterion (AIC) (38). The degrees of freedom corresponding to the date, daily average temperature, daily average relative humidity, atmospheric pressure, and wind speed are 7, 6, 3, 3, and 5, respectively (21, 26, 39).

After establishing the basic model, the pollutant concentration data were introduced to estimate the regression coefficient *β* for the air pollutants. The relative risks (RR) and their 95% confidence intervals (CI) were calculated for each 10 μg/m^3^ (NO_2_, O_3_, PM_10_, PM_2.5_, SO_2_) or 1 mg/m^3^ (CO) increase in pollutant concentrations, using the following equations Equations (2,3):


(2) RR=exp(β×ΔC)

(3) RR(95%CI)=exp(ΔC×(β±1.96SE))

To identify potential high-risk groups and to further consider the effects of temperature, subgroup analyses were carried out for gender (male and female), age (65–69 years, ≥70 years), and seasonal factors (warm season: April to September; cold season: October to March), respectively. We analyzed each subgroup using a single-pollutant model, and then selected the exposure response coefficients of the maximum impact estimates in the single-pollutant model for comparison. Differences in effect estimates between subgroups were assessed using *Z* tests using the following equation (40) Equation (4):


(4) $$Z=\left(\hat{Q_{1}}-\hat{Q_{2}}\right)/\sqrt{{\hat{SE}}_{1}^{2}+{\hat{SE}}_{2}^{2}}$$

where $\hat{Q_{1}}$ and $\hat{Q_{2}}$ are the RR estimates for different categories in each subgroup (e.g., male and female), and ${\hat{SE}}_{1}^{2}$ and ${\hat{SE}}_{2}^{2}$ are their respective standard deviations.

All statistical analyses in this study were conducted using R4.2.0, and the quasi-Poisson regression model was constructed using the “mgcv” and “splines” packages. The statistical tests were two-sided, and associations with *p* < 0.05 were considered statistically significant.

#### 2.3.2 Economic costs of asthma hospitalization attributable to air pollution

In general, exceeding air quality guideline levels is associated with significant risks to health. Since the concentrations of pollutants CO, O_3_and SO_2_ at lag0 to lag7 days are far lower than the WHO air quality standard (CO 24 h mean value is 4 mg/m^3^, O_3_ 8 h mean value is 100 μg/m^3^, SO_2_ 24 h mean value is 40 μg/m^3^) (41). Therefore, we did not measure the burden related to CO, O_3_ and SO_2_. Although previous research suggests that most of the burden from air pollution is attributed to PM pollution, the effects of NO_2_ cannot be ignored (42). Based on the expose-response coefficient analyzed by GAM model, we included NO_2_, PM_2.5_ and PM_10_ whose pollutant concentrations exceeded the reference standard (24 h averages for NO_2_ of 25 μg/m^3^, PM_10_ of 45 μg/m^3^, and PM_2.5_ of 15 μg/m^3^) into the burden analysis range. We used the attributable risk method to calculate the number of HAs for asthma patients over 65 years old due to exposure to air pollutants (40, 43), the formulae are as follows Equations (5,6):


(5) $$AR_{i}=\left(\exp\left(\beta_{s}\times \Delta AP_{i}\right)-1\right)/\exp\left(\beta_{s}\times \Delta AP_{i}\right)$$

(6) $$\mathrm{AN}=\overset{1076}{\underset{i=1}{\sum }}\left({\mathrm{AR}}_{i}\times N_{i}\right)$$

where i is the number of days in the study period (from 1 to 1,076). $\beta_{s}$ is equal to the sum of the *β* coefficients of the lag days with significant effects (for example: the effect of PM_2.5_ is significant only from lag5 to lag7, and the coefficient *βs* is equal to the sum of the coefficients from lag5 to lag7) (44, 45). In this study, the β values of PM_2.5_, PM_10_ and NO_2_ were 0.0044, 0.0041and 0.0043, respectively. ${\mathrm{AR}}_{i}\;$ is the attributable risk on day i . $\Delta {\mathrm{AP}}_{i}$ is the difference between the observed concentrations of pollutants on day i and the reference concentrations. $N_{i}$ is the number of asthma hospitalizations on day i . AN is the total number of asthma inpatients attributable to air pollution.

The next, based on the AN , we used the cost of illness method to estimate the economic cost of HAs for asthma due to PM and NO_2_ exposure, which involved with direct medical cost and indirect economic cost (40, 46) Equations (7,8).


(7) $$\mathrm{DC}=\mathrm{AN}\times {\mathrm{Cost}}_{\mathrm{total}}$$

(8) $$\mathrm{IC}=\mathrm{AN}\times \mathrm{dPCDI}\times \mathrm{mean}H_{d}$$

DC is the direct medical cost attributable to air pollution. ${\mathrm{Cost}}_{\mathrm{total}}$ is the average direct medical cost per patient during the study period. IC is the indirect economic cost attributable to air pollution. $\mathrm{mean}H_{d}$ is the average number of hospitalization days per case. dPCDI is the per capital daily disposable income of residents in Sichuan province, since our research object is the people over 65 years old, most of whom may be in the state of retirement, we introduced the Labour force participation rate (47) of people aged 65 years or over of China reported by the International Labor Organization^3^ to adjust dPCDI .

### 2.4 Sensitivity analysis

First, according to the lag day corresponding to the maximum adverse effect in the single-pollutant models, the pollutants at the corresponding lag days were introduced into the model individually to enable the fitting of two-pollutant models. To avoid collinearity, air pollutants with a correlation coefficient *r* > 0.60 were excluded from the multi-pollutant model. Second, the model was fitted by varying the degrees of freedom of the temporal trends (df = 5–9) to assess temporal stability (48).

## 3 Results

### 3.1 Basic situation

From January 1, 2017 to December 31, 2019, a total of 6,092 older adults asthma patients were admitted to the participating hospitals. Among them, 39.3% (*n =* 2,394) were male, and 60.7% (*n* = 3,698) were female. Meanwhile, 36.10% (*n =* 2,199) of the patients were aged 65–69 years, while 63.90% (*n =* 3,893) were aged ≥70 years. Compared with the warm season, slightly more patients (58.32%, *n* = 3,553) were admitted during the cold season.

### 3.2 Statistical description of pollutants and meteorological variables during the study period

The daily average concentration of CO was 0.80 mg/m^3^, NO_2_, O_3_, PM_10_, PM_2.5_, and SO_2_ during the study period were 28.72, 77.26, 72.07, 47.40, and 11.46 μg/m^3^, respectively. The average daily temperature and relative humidity were 16.17°C and 77.90%, and the average daily atmospheric pressure was 956.78 Pa, with an average wind speed of 1.73 M/s. These data are detailed in Table 1.

**Table 1:** Statistical description of air pollutants and meteorological variables in the 9 cities of Sichuan province, 2017–2019.

Pollutants	Mean (SD)	Minimum	P_25_	P_50_	P_75_	Maximum
**Air pollutant concentration (mg/m** ^**3**^ **or μg/m** ^**3**^ **)**						
CO	0.80 (0.27)	0.11	0.62	0.77	0.95	9.17
NO_2_	28.72 (14.30)	2.09	18.29	25.86	36.14	127.10
O_3_	77.26 (39.62)	1.64	47.85	70.13	99.91	287.96
PM_10_	72.07 (44.37)	3.06	39.09	60.46	94.51	441.48
PM_2.5_	47.40 (32.96)	3.06	23.46	38.05	62.38	269.35
SO_2_	11.46 (6.03)	1.07	7.62	10.02	13.47	91.22
**Meteorological factors**						
Temperature (°C)	16.17 (7.45)	−0.92	9.33	16.16	22.62	33.82
Relative humidity (%)	77.90 (11.75)	15.31	70.34	79.41	86.89	99.97
Pressure (Pa)	956.78 (25.81)	656.72	950.93	959.96	970.54	1002.93
Wind speed (m/s)	1.73 (0.63)	0.02	1.33	1.63	2.00	10.03

SD, standard deviation.

### 3.3 Correlations between air pollutants and meteorological factors

The results of the Spearman correlation analysis between air pollutants and meteorological factors are shown in Supplementary Table S1. We can observe significant positive correlation between CO and NO_2_, PM_10_, PM_2.5_ and SO_2_, with the correlation coefficient ranging from 0.4006 to 0.7464, and the correlation coefficient *r* between CO and O_3_ is −0.2311. NO_2_ and PM_10_, PM_2.5_ and SO_2_ with the correlation coefficient ranging from −0.172 to 0.7252. In addition, O_3_ is negatively correlated with PM_10_ and PM_2.5_. PM_10_ is positively correlated with PM_2.5_ and SO_2_. The correlation coefficient *r* between PM_2.5_ and SO_2_ is 0.4902. The temperature was significantly negatively correlated with all six pollutants, while relative humidity was significantly negatively correlated with the remaining five pollutants excluding CO. Atmospheric pressure was negatively correlated with O_3_ and positively correlated with CO, PM_10_, and PM_2.5_, while its correlation with NO_2_ was not significant. Wind speed was negatively correlated with CO, NO_2_, PM_10_, and PM_2.5_, and positively correlated with O_3_ and SO_2_.

### 3.4 Relationship between air pollution and risk of hospitalization for asthma

**Figure 2:**
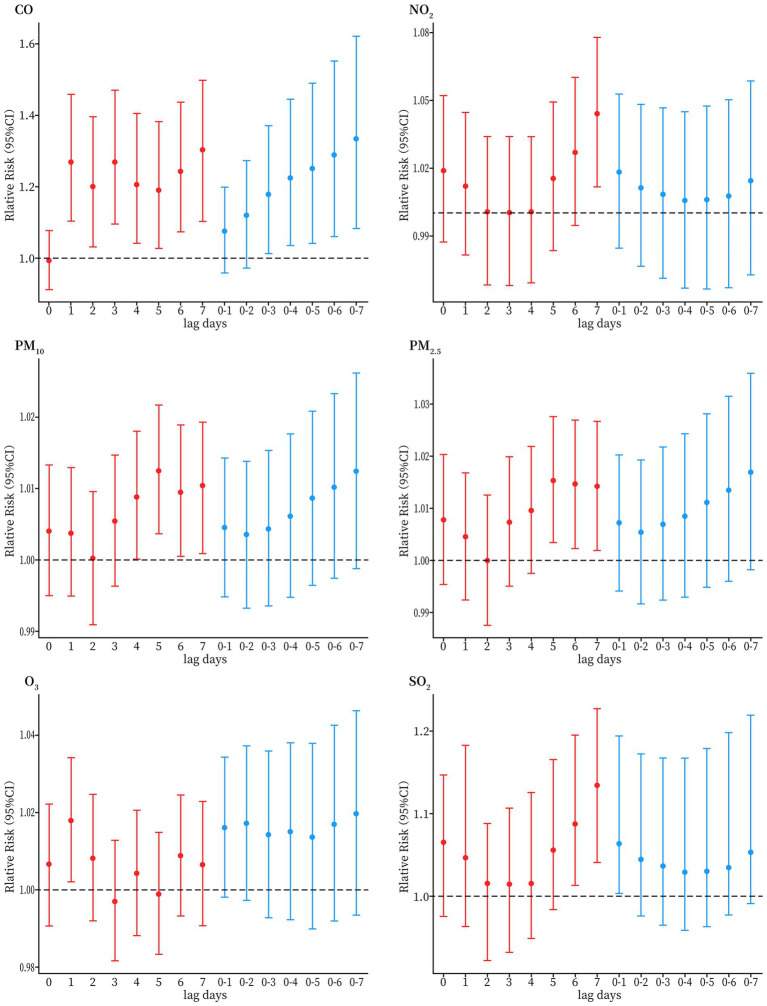
Relative risks (95% CI) of older adults HAs for asthma per 10 μg/m^3^ increase in concentrations of air pollutants (1 mg/m^3^ increase in CO) for different lag days in the single pollutant models in Sichuan province, China, during 2017–2019.

The results of the single-pollutant model analysis indicated that short-term exposure to pollutants was positively associated with asthma hospitalization among the older adults. According to the single-day lag (lag0–lag7) results, CO exposure increased the risk of asthma hospitalization in the older adults except at lag0, and the maximum risk was identified at lag7 with an RR of 1.327 (95% CI:1.116–1.577). There is obvious association of NO_2_ with hospitalization for asthma at lag 7, with an RR of 1.044 (95% CI: 1.011–1.078). PM_10_ increased the risk of asthma hospitalization among the older adults at lag4, lag5, lag6, and lag7, and the adverse effects of PM_2.5_ exposure were observed at lag5, lag6, and lag7, the maximum RR values were observed at lag5 for both PM_10_ and PM_2.5_, being 1.013 (95% CI: 1.004–1.022) and 1.015 (95% CI: 1.003–1.028), respectively. The effect of O_3_ on asthma hospitalization at lag1, with an RR value of 1.018 (95% CI: 1.002–1.034). And SO_2_ increased hospitalization risk for asthma at lag1, lag6, and lag7 with an RR value of 1.13 (95% CI: 1.041–1.227) at lag7. Among the moving average multi-day exposure (lag01–lag07) results, CO had a significant effect on asthma hospitalizations at lag03, lag04, lag05, lag06, and lag07, with a maximum RR of 1.372 (95% CI: 1.095–1.72) at lag07. SO_2_ had a significant effect on asthma hospitalizations at lag01, with a maximum RR of 1.094 (95% CI: 1.002–1.195). These results are shown in Figure 2 and Supplementary Table S2.

### 3.5 Stratified analysis

**Figure 3:**
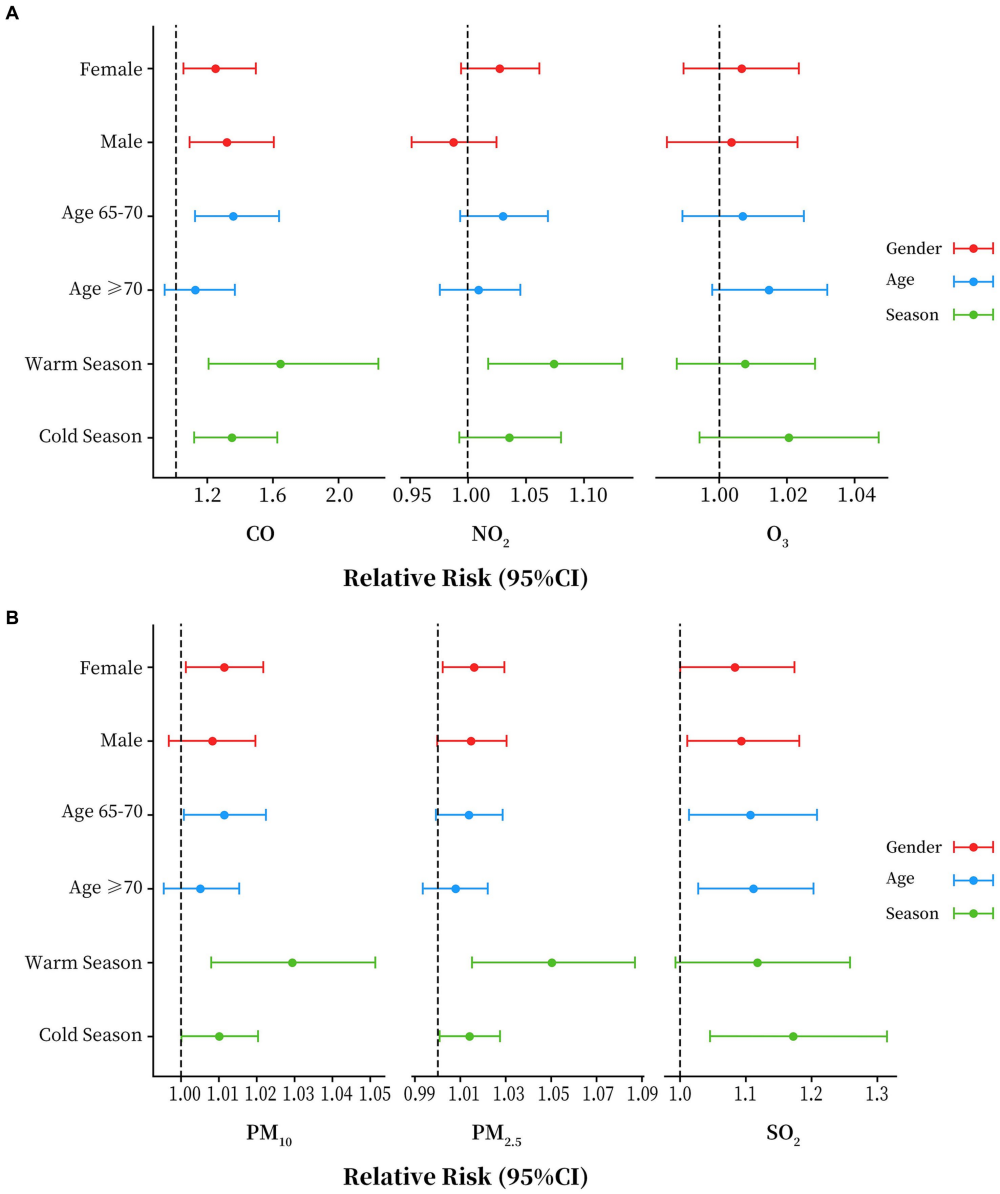
Stratified analyses by age, season and gender for each air pollutant in Sichuan province, China, 2017 to 2019. **(A)** Results of stratified analysis of CO, NO_2_ and O_3_. **(B)** Results of stratified analysis of PM_10_, PM_2.5_ and SO_2_.

In gender stratification, the effects of CO, PM_2.5_, PM_10_, and SO_2_, exposure were significant on female, only CO and SO_2_ had significant effects on male, but the difference between the genders was not statistically significant (*p* > 0.05). In age stratification, CO and PM_10_ have a significant impact on the population aged 65–69, however, we did not observe differences in the effects of air pollutants on different age groups (*p* > 0.05). As for the effects in different seasons, CO, NO_2_, PM_10_ and PM_2.5_ had positive and significant effects on asthma HAs in the warm season, but the difference between the cold and warm seasons was not statistically significant (*p* > 0.05) as shown in Figure 3 and Supplementary Table S3.

### 3.6 Economic costs attributable to air pollution

Table 2 listed the attributable number of HAs and the related economic cost due to exceeding PM and NO_2_ exposure involving WHO air quality standard in Sichuan province 2017–2019. Based on the reference concentrations, 764, 581 and 95 total cases of HAs for asthma could be attributable to PM_2.5_, PM_10_ and NO_2_, separately. The total economic cost caused by exposure to particulate matter (PM, includes PM_2.5_ and PM_10_) was 10.952 million CNY, including a direct medical cost of 10.788 million CNY, and an indirect economic cost of 0.164 million CNY. Exposure to PM_2.5_ caused the highest economic cost of 6.222 million CNY, while that attributable to NO_2_ exposure was 0.776 million CNY.

**Table 2:** The attributable number of hospitalizations and economic cost due to pollution in Sichuan province, 2017–2019.

Variable	PM_2.5_	PM_10_	NO_2_	Total
AN^a^	764 (146–1,274)	581 (87–956)	95 (26–157)	1,440
AR^b^	0.133 (0.024–0.229)	0.106 (0.015–0.189)	0.017 (0.004–0.029)	—
DC^c^^,^^e^	6.129 (1.169–10.214)	4.659 (0.696–7.669)	0.764 (0.212–1.258)	11.552
IC^d^^,^^e^	0.093 (0.018–0.155)	0.071 (0.011–0.117)	0.012 (0.003–0.019)	0.176
Costs^e^	6.222	4.730	0.776	11.727

a Attributable number of hospitalizations.

b AR, attributable risk.

c DC, direct medical cost of hospitalizations attributable to pollution.

d IC, indirect economic cost of hospitalizations attributable to pollution.

e Unit: million CNY.

### 3.7 Sensitivity analysis

In our sensitivity analyses, we found that the associations between air pollution exposure and asthma exacerbation remained almost unchanged in magnitude and remained statistically significant. Upon introducing other pollutants one by one into the single-pollutant model for adjustment, the effect of the original pollutant was somewhat affected. The effects of some pollutants were weakened. For example, the inclusion of NO_2_, O_3_ and SO_2_ reduced the RR of hospitalization for asthma attributable to CO exposure among the older adults, but the effect of CO remained significant. Conversely, the inclusion of O_3_ in analyses increased the risk of hospitalization for asthma attributable to particulate matter among the older adults. Overall, except for O_3_ and SO_2_, there was little change in the effect of the primary pollutant on asthma admissions in older adults after the introduction of another pollutant. This suggests a strong correlation between pollutants, and that the effects of air pollutants on asthma are not simply additive or subtractive, but that complex antagonistic or synergistic effects between pollutants may exist (49, 50). These results are shown in Supplementary Table S4. When the temporal degrees of freedom varied from 5 to 9, no significant changes were observed in the daily asthma hospitalization rate for each 10 μg/m^3^ increase in air pollutant concentrations (1 mg/m^3^ increase in CO), indicating good model stability. The results are shown in Supplementary Table S5.

## 4 Discussion

Our study is one of the few multi-urban, multi-pollutant studies in heavily polluted areas in China, and the results enrich the empirical evidence of the positive association between short-term exposure to air pollution and asthma hospitalization among the older adults (51). Taking into consideration controlling factors such as temperature, humidity, atmospheric pressure, wind speed, and holiday effects, our study identified positive associations between short-term exposure to air pollution and the risk of hospitalization for asthma among the older adults, and the economic cost of asthma hospitalizations attributable to air pollution during the study period was calculated to be 11.727 million CNY in total during the study period, 2017–2019.

In the single-pollutant model, when the concentrations of pollutants increased by 10 ug/m^3^ (1 mg/m^3^ increase in CO), there were positive associations between CO, NO_2_, SO_2_, and O_3_, and the hospitalizations of older adult with asthma, with an RR of 1.327 (95% CI:1.116–1.577), 1.044 (95% CI: 1.011–1.078), 1.13 (95% CI: 1.041–1.227) and 1.018 (95% CI, 1.002–1.034), respectively, demonstrating a lag effect in the impact of air pollutants on asthma, which was consistent with previous researches. For CO, a study in Dongguan (52) reported that the per interquartile range (IQR) increase in ambient CO at lag03 day corresponded to 8.86% (95% CI, 4.89, 12.98%) increased risk in outpatient visits for asthma. This may be because exposure to CO can lead to tissue hypoxia to cause damage and can promote the development of airway inflammatory diseases (53, 54). As irritating air pollutants, NO_2_ and SO_2_ also have significant effects on respiratory health (55). The study of Raji et al. (25) from Iran Ahwaz discovered that each 10 μg/m^3^ increase in NO_2_, and SO_2_ concentrations was associated with an increase of 6.9 and 6.9% in the risk of hospitalization for asthma in people aged ≥60 years, respectively. NO_2_ is associated with the production of various free radicals that can trigger lipid peroxidation of cell membranes, and these effects can damage the structure and impair airway function, and exposure to NO_2_ can also promote the release of inflammatory mediators, leading to asthma (56, 57). Such as Stosic et al. (58) identified a 1.2% increase in the RR of daily asthma hospitalization among people aged ≥65 years in association with a 10 μg/m^3^ increase in daily NO_2_ concentration. Similarly, SO_2_ has been previously shown to cause bronchospasm and to increase the risk of hospitalization for asthma (18, 28). O_3_ is a highly oxidizing and reactive gas, which has been associated with a variety of adverse respiratory outcomes (59). Previous epidemiological studies have investigated the association between ozone pollution and acute asthma exacerbations (60, 61). For example, a study in Hong Kong (62) reported that an interquartile range increment in O_3_ (31.6 μg/m^3^) in a previous week (lag0-6) was associated with 13.2% (8.4%–18.2%) increases in asthma for elders. Although the associations between air levels and asthma admissions are different due to variations in regions, population, and social factors and so on, these epidemiological studies all support that the risk of asthma increases with the concentration of air pollutants.

PM is a complex mixture of solid and liquid particles suspended in the atmosphere, and includes PM_10_ (≤10 μm) and PM_2.5_ (≤2.5 μm). The former is mainly produced by construction activities and re-suspension of road dust and wind, while the latter arises mainly from combustion processes. Increasing studies reported the positive associations between PM pollution and HAs for asthma among older adults (24–26). For example, Park et al. (63) using adults as the referent, the relative rate (RR) of asthma admissions with 10 μg/m^3^ increase of PM_10_ is 1.3% (95% CI 0.7–1.9%) higher for the the people over 65 years old. Xie et al. (64) found that the older adults were more susceptible to PM_10_, with a cumulative RR of 1.066 (1.015–1.119) for hospitalizations at lag12, but our results indicate that PM_2.5_ is significantly associated with admission to hospital for asthma in the older adults, and RR at lag5 is 1.015 (1.003–1.028). The composition of PM may vary depending on the area where PM is generated, the season, and weather conditions (65). Upon entering the respiratory tract, PM deposits to exert toxic effects through mechanical damage and harmful substances on the surface, which cause pathological changes such as mucosal edema, epithelial cell proliferation, vasodilation, and eosinophilic infiltration, which lead to or aggravate airway inflammation and oxidative stress (66, 67), thus causing asthma. All of the above-mentioned processes play pivotal roles in the occurrence of asthma. However, this evidence comes primarily from observational studies, so future epidemiological studies and *in vivo* assays based on more precise environmental exposure data are needed to explore the biological mechanisms of air pollutants under the premise of better controls for confounding factors.

The identification of potentially susceptible populations plays a significant role in public health. In the stratified analysis, we observed stronger associations between air pollution and asthma HAs among older adult in females, those aged 65–69 years, and in the warm season, although all of the differences between subgroups did not reach statistical significance. Previous studies have reported stronger respiratory-related health outcomes in females compared to males (68, 69). Similar to our findings, a study conducted in Canadian found that only in females, short-term NO_2_ exposure was associated with increased risk of respiratory hospitalizations (70). As for the effects of the season, studies have shown a higher correlation between individual air pollutant exposure and ambient air pollutant concentrations in summer than in winter (71), which makes our findings somewhat reasonable. The above discussion highlights some potential reasons why the associations between air pollution and asthma among older adults in this paper may differ from gender, season. However, our results were inconsistent across pollutants, making it more difficult to explain the biological or physiological mechanisms of effects. In order to better understand the health hazards of air pollution in older adults, future studies need to focus on more information such as individual activity patterns, occupations and regional economy and development situation, etc.

Finally, the present study analyzed the burden attributable to air pollution-induced asthma hospitalizations, which is central for cost-effective policy-making and asthma prevention. Assessing the corresponding economic burden of exposure to pollutants that exceed the limits also can shed more light on the potential link between air pollution and health. Previous studies have proved that PM and NO_2_ cause serious economic losses to the society (45, 72, 73). For example, Moradi et al. (74) found that in conditions of PM_2.5_ concentration above 5 μg/m^3^, attributed proportion, the total number of attributable cases, and the number of attributable cases per 100,000 population (with moderate relative risk and confidence of 95%) for the admission of respiratory diseases have been estimated at 97.1%, 68 persons and 3 persons, respectively. Guo et al. (20) assessed the economic burden of medical visits for air pollution-induced asthma in Shanghai in 2014, and the economic costs attributable to PM_10_, PM_2.5_, and NO_2_ were 30.18, 34.50, and 17.15 million USD annually, respectively. Gao et al. (75) evaluated the economic costs associated with the haze event of January 2013 and found that PM_2.5_ caused asthma-related economic costs of 7.1 million USD. These studies all show a positive correlation between air pollution and burden. Meanwhile, we found that the number of asthma hospitalizations attributable to PM_2.5_, PM_10_, and NO_2_ pollution was 749, 409 and 101, respectively, which resulted in a total economic cost of 5.94 million CNY, 3.5 million CNY and 0.63 million CNY, respectively. These findings suggest that more hospitalizations and economic lost could be avoided if recorded PM and NO_2_ levels is at lower levels. Our study only assessed people over 65 years of age, which is different from other population-wide assessments, despite this, it cannot be ignored that the impact of air pollution on the health of the older adults population in Sichuan remains a more serious public health challenge compared with other regions.

### 4.1 Strengths and limitations

This study has made two contributions. First, data were collected from nine cities and prefectures across Sichuan province, which can represent the overall characteristics of the province to some extent. Second, the relationships between air pollutants and asthma hospitalization among the older adults were examined and discussed, and the economic burden was assessed, providing empirical evidence for the association between air pollutants and asthma among the older adults. Meanwhile, this study has certain limitations. First, this is an ecological study, although it was adjusted for several confounding factors such as the day of the week, public holidays, and weather conditions, asthma has multiple etiologic factors, and the interference and influence of confounding factors such as environmental chemicals, personal health status, and individual lifestyle habits were unavoidable in this study. Second, the air pollutant concentration data were obtained from fixed monitoring sites and measurement bias of exposure levels was inevitable. Finally, in calculating the economic costs, we may be underestimating the burden of air pollution because we only consider the number of people treated in hospital and use the number of days in hospital to measure lost productivity.

Based on these findings, first, strategies for prevention of asthma should focus on providing educational health messages in older adults (≥65). Second, because there is no completely safe level of air pollutants, more stringent regulations on industry and automobiles, and a more active notification system for high levels of air pollutants, may help to prevent asthma. Third, reduced air pollution from industrial upgrades, vehicle and fuel renovations, better public transportation and increasing green space (76) can prevent respiratory diseases. Finally, we need a comprehensive pre-warning system to help patients with asthma diseases prepare and take preventive measures in advance, and reduce the social and economic burden of air pollution.

## 5 Conclusion

We found that short-term exposure to air pollutants was significantly associated with increased the risk of asthma hospitalization in people aged ≥65 years with a lag effect. Additionally, the number of asthma hospitalizations attributable to PM and NO_2_ pollution during the study period was 1,345 and 95, respectively, with a total economic cost of 10.952 and 0.776 million CNY, respectively. These findings are of great importance to the formulation of health care policies for the older adult population and the direction of public health development.

## Data availability statement

The original contributions presented in the study are included in the article/Supplementary material, further inquiries can be directed to the corresponding authors.

## Author contributions

YZ: Data curation, Formal analysis, Methodology, Writing – original draft, Writing – review & editing. XY: Writing – review & editing. WJ: Methodology, Writing – review & editing. XG: Methodology, Writing – review & editing. BY: Methodology, Writing – review & editing. XF: Supervision, Writing – review & editing. LY: Data curation, Funding acquisition, Investigation, Methodology, Supervision, Writing – review & editing.

## Footnotes

^1^ http://www.scdata.net.cn/

^2^ https://lbs.amap.com/

^3^ https://www.ilo.org/global/lang--en/index.htm
