# Supplementary material for: Short-term effects of air pollutants on hospital admissions for asthma among older adults: a multi-city time series study in Southwest, China
Source: Front Public Health. 2024 Jan 29;12:1346914. doi: 10.3389/fpubh.2024.1346914 (PMC10859495; doi:10.3389/fpubh.2024.1346914)
Supplement: Supplementary file 1 [file Table_1.DOCX]

**Supplementary Information for**

**Short-term effects of air pollutants on hospital admissions for asthma among older adults: a multi-city time series study in Southwest, China**

Yuqin Zhang^1^, Xi Yang^1^,XingLin Feng^2*^, Lian Yang^1*^, Wanyanhan Jiang^1^, Xi Gao^1^ and Biao Yang^1^

# Authors' information

1 School of Public Health, Chengdu University of Traditional Chinese Medicine, Chengdu 610075, Sichuan, China.

2 School of Public Health,Peking University,Peking 100871,China.

* Corresponding author :

XingLin Feng, Email:fxl@bjmu.edu.cn

Lian Yang, Email:yyanglian@163.com

Telephone：+86 13540359258

Fax numbers (with country and area code) ：028-61800000

**Supplementary Tables**

**Supplementary Table 1.**Spearman correlation coefficients between daily meteorological factors and air pollutants for Sichuan (2017–2019)

| **\|Indicator** | **CO** | **NO_2_** | **O_3_** | **PM_10_** | **PM_2.5_** | **SO_2_** | **temperature** | **humidity** | **pressure** | **wind-speed** |
| --- | --- | --- | --- | --- | --- | --- | --- | --- | --- | --- |
| CO | 1 |  |  |  |  |  |  |  |  |  |
| NO_2_ | 0.5427  （<.001) | 1 |  |  |  |  |  |  |  |  |
| O_3_ | -0.2311  （<.001) | -0.172  （<.001) | 1 |  |  |  |  |  |  |  |
| PM_10_ | 0.6949  （<.001) | 0.7252  （<.001) | -0.1363  （<.001) | 1 |  |  |  |  |  |  |
| PM_2.5_ | 0.7464  （<.001) | 0.6957  （<.001) | -0.2155  （<.001) | 0.9691  （<.001) | 1 |  |  |  |  |  |
| SO_2_ | 0.4006  （<.001) | 0.4866  （<.001) | 0.0075  （.81) | 0.5086  （<.001) | 0.4902  （<.001) | 1 |  |  |  |  |
| temperature | -0.445  （<.001) | -0.4436  （<.001) | 0.7143  （<.001) | -0.5165  （<.001) | -0.5634  （<.001) | -0.1925  （<.001) | 1 |  |  |  |
| humidity | -0.0654  （.03) | -0.2337  （<.001) | -0.5471  （<.001) | -0.364  （<.001) | -0.2682  （<.001) | -0.3526  （<.001) | -0.0842  （.005) | 1 |  |  |
| pressure | 0.2095  （<.001) | 0.2224  （<.001) | -0.4309  （<.001) | 0.2963  （<.001) | 0.324  （<.001) | 0.0835  （.006) | -0.5664  （<.001) | 0.1286  （<.001) | 1 |  |
| wind-speed | -0.1486  （<.001) | -0.1078  （<.001) | 0.1925  （<.001) | -0.0931  （<.002) | -0.112  （<.001) | 0.0674  （.03) | 0.1481  （<.001) | -0.1522  （<.001) | -0.1867  （<.001) | 1 |

**Supplementary Table 2.**Relative risks (95% confidence interval) of elderly hospital admissions for asthma per 10 μg/m^3^ increase in concentrations of air pollutants (1mg/m^3^ increase in CO) for different lag days in the single pollutant models in Sichuan Province, China, during 2017–2019.

| **Lag days** | **CO** | |  | **NO_2_** | |  | **O_3_** | |  | **PM_10_** | |  | **PM_2.5_** | | | |  | | **SO_2_** | | | |  |
| --- | --- | --- | --- | --- | --- | --- | --- | --- | --- | --- | --- | --- | --- | --- | --- | --- | --- | --- | --- | --- | --- | --- | --- |
|  | **RR** | **95%CI** |  | **RR** | **95%CI** |  | **RR** | **95%CI** |  | **RR** | **95%CI** |  | **RR** | | **95%CI** | |  | | **RR** | | **95%CI** | |  |
| Lag_0 | 0.991 | 0.9-1.09 |  | 1.019 | 0.987-1.052 |  | 1.006 | 0.991-1.022 |  | 1.004 | 0.995-1.013 |  | | 1.008 | | 0.995-1.02 | |  | | 1.057 | | 0.975-1.146 | |
| Lag_1 | 1.312 | 1.121-1.535 |  | 1.012 | 0.981-1.045 |  | 1.018 | 1.002-1.034 |  | 1.004 | 0.995-1.013 |  | | 1.005 | | 0.992-1.017 | |  | | 1.092 | | 1.008-1.183 | |
| Lag_2 | 1.228 | 1.032-1.462 |  | 1.001 | 0.968-1.034 |  | 1.008 | 0.992-1.025 |  | 1 | 0.991-1.01 |  | | 1 | | 0.988-1.013 | |  | | 1.001 | | 0.921-1.087 | |
| Lag_3 | 1.308 | 1.106-1.546 |  | 1 | 0.968-1.034 |  | 0.997 | 0.981-1.013 |  | 1.006 | 0.996-1.015 |  | | 1.007 | | 0.995-1.02 | |  | | 1.015 | | 0.932-1.106 | |
| Lag_4 | 1.239 | 1.048-1.466 |  | 1.001 | 0.969-1.034 |  | 1.004 | 0.988-1.021 |  | 1.009 | 1-1.018 |  | | 1.01 | | 0.997-1.022 | |  | | 1.033 | | 0.948-1.125 | |
| Lag_5 | 1.219 | 1.029-1.444 |  | 1.016 | 0.984-1.049 |  | 0.999 | 0.983-1.015 |  | 1.013 | 1.004-1.022 |  | | 1.015 | | 1.003-1.028 | |  | | 1.07 | | 0.983-1.165 | |
| Lag_6 | 1.276 | 1.08-1.507 |  | 1.027 | 0.994-1.06 |  | 1.009 | 0.993-1.025 |  | 1.01 | 1.001-1.019 |  | | 1.015 | | 1.002-1.027 | |  | | 1.1 | | 1.013-1.194 | |
| Lag_7 | 1.327 | 1.116-1.577 |  | 1.044 | 1.011-1.078 |  | 1.007 | 0.991-1.023 |  | 1.01 | 1.001-1.019 |  | | 1.014 | | 1.002-1.027 | |  | | 1.13 | | 1.041-1.227 | |
| Lag_01 | 1.081 | 0.948-1.232 |  | 1.018 | 0.985-1.053 |  | 1.016 | 0.998-1.034 |  | 1.005 | 0.995-1.014 |  | 1.007 | | 0.994-1.02 | |  | | 1.094 | | 1.002-1.195 | |  |
| Lag_02 | 1.128 | 0.965-1.319 |  | 1.012 | 0.976-1.048 |  | 1.017 | 0.997-1.038 |  | 1.004 | 0.993-1.014 |  | 1.005 | | 0.992-1.019 | |  | | 1.07 | | 0.976-1.172 | |  |
| Lag_03 | 1.204 | 1.012-1.433 |  | 1.008 | 0.971-1.047 |  | 1.014 | 0.993-1.036 |  | 1.004 | 0.994-1.015 |  | 1.007 | | 0.992-1.022 | |  | | 1.061 | | 0.964-1.167 | |  |
| Lag_04 | 1.255 | 1.039-1.516 |  | 1.005 | 0.967-1.045 |  | 1.015 | 0.992-1.038 |  | 1.006 | 0.995-1.018 |  | 1.008 | | 0.993-1.024 | |  | | 1.057 | | 0.958-1.167 | |  |
| Lag_05 | 1.281 | 1.046-1.569 |  | 1.006 | 0.966-1.048 |  | 1.014 | 0.99-1.038 |  | 1.009 | 0.997-1.021 |  | 1.011 | | 0.995-1.028 | |  | | 1.066 | | 0.963-1.18 | |  |
| Lag_06 | 1.324 | 1.068-1.642 |  | 1.008 | 0.967-1.05 |  | 1.017 | 0.992-1.043 |  | 1.01 | 0.997-1.023 |  | 1.014 | | 0.996-1.031 | |  | | 1.081 | | 0.975-1.198 | |  |
| Lag_07 | 1.372 | 1.095-1.72 |  | 1.015 | 0.973-1.059 |  | 1.02 | 0.994-1.047 |  | 1.012 | 0.999-1.026 |  | 1.017 | | 0.998-1.036 | |  | | 1.099 | | 0.991-1.219 | |  |

**Supplementary Table 3.** The Relative risks (95% CI) of age, season, and sex group in overall HAs for asthma linking with a 10 μg/m^3^ increase in pollutant concentrations (1 mg/m^3^ increase in CO) in Sichuan Province in 2017-2019.

| **Variables** | **CO** | | | **NO_2_** | | |
| --- | --- | --- | --- | --- | --- | --- |
|  | **RR(95%CI)** | ***Z* value** | ***P* value** | **RR(95%CI)** | ***Z* value** | ***P* value** |
| **Gender** |  |  |  |  |  |  |
| Female | 1.248(1.044-1.491) | 0.39 | 0.69 | 1.027(0.994-1.062) | 1.55 | 0.12 |
| Male | 1.316(1.08-1.604) |  |  | 0.988(0.951-1.025) |  |  |
| **Age** |  |  |  |  |  |  |
| 65-69 | 1.351(1.112-1.64) | 1.33 | 0.18 | 1.031(0.994-1.069) | 0.78 | 0.43 |
| ≥70 | 1.121(0.925-1.36) |  |  | 1.01(0.976-1.045) |  |  |
| **Season** |  |  |  |  |  |  |
| Warm season | 1.642(1.195-2.257) | 1.05 | 0.29 | 1.074(1.017-1.134) | 1.04 | 0.30 |
| Cold season | 1.345(1.109-1.631) |  |  | 1.036(0.993-1.08) |  |  |
| **Variables** | **PM_10_** | | | **PM_2.5_** | | |
|  | **RR(95%CI)** | ***Z* value** | ***P* value** | **RR(95%CI)** | ***Z* value** | ***P* value** |
| **Gender** |  |  |  |  |  |  |
| Female | 1.011(1.001-1.022) | 0.42 | 0.67 | 1.016(1.002-1.029) | 0.07 | 0.94 |
| Male | 1.008(0.997-1.02) |  |  | 1.015(0.09996-1.03) |  |  |
| **Age** |  |  |  |  |  |  |
| 65-69 | 1.012(1.001-1.022) | 0.83 | 0.41 | 1.014(0.999-1.028) | 0.59 | 0.55 |
| ≥70 | 1.005(0.995-1.015) |  |  | 1.007(0.993-1.022) |  |  |
| **Season** |  |  |  |  |  |  |
| Warm season | 1.029(1.008-1.051) | 1.59 | 0.11 | 1.05(1.015-1.086) | 1.88 | 0.06 |
| Cold season | 1.01(0.99993-1.02) |  |  | 1.014(1.001-1.027) |  |  |
| **Variables** | **O_3_** | | | **SO_2_** | | |
|  | **RR(95%CI)** | ***Z* value** | ***P* value** | **RR(95%CI)** | ***Z* value** | ***P* value** |
| **Gender** |  |  |  |  |  |  |
| Female | 1.006(0.989-1.023) | 0.20 | 0.84 | 1.084(1.001-1.174) | 0.15 | 0.88 |
| Male | 1.004(0.984-1.023) |  |  | 1.094(1.012-1.182) |  |  |
| **Age** |  |  |  |  |  |  |
| 65-69 | 1.007(0.989-1.025) | 0.61 | 0.54 | 1.107(1.014-1.209) | 0.08 | 0.94 |
| ≥70 | 1.015(0.998-1.032) |  |  | 1.112(1.028-1.203) |  |  |
| **Season** |  |  |  |  |  |  |
| Warm season | 1.008(0.987-1.028) | 0.74 | 0.46 | 1.118(0.994-1.258) | 0.57 | 0.57 |
| Cold season | 1.02(0.994-1.047) |  |  | 1.173(1.046-1.315) |  |  |

**Supplementary Table 4.** RRs and 95% CIs for asthma hospitalization associated with a 10 μg/m^3^ increase in pollutant concentrations (1 mg/m^3^ increase in CO) in single and two-pollutant models.

| Variables | Two-pollutant model | RR | 95% CI | **Lag days** |
| --- | --- | --- | --- | --- |
| CO |  | 1.372 | 1.095-1.72 | Lag 07 |
|  | Adjusted for NO_2_ | 1.342 | 1.053-1.711 | Lag 07 |
|  | Adjusted for O_3_ | 1.358 | 1.073-1.718 | Lag 07 |
|  | Adjusted for SO_2_ | 1.317 | 1.039-1.669 | Lag 07 |
| NO_2_ |  | 1.044 | 1.011-1.078 | Lag 7 |
|  | Adjusted for CO | 1.032 | 0.993-1.072 | Lag 7 |
|  | Adjusted for O_3_ | 1.055 | 1.02-1.091 | Lag 7 |
|  | Adjusted for SO_2_ | 1.045 | 1.009-1.082 | Lag 7 |
|  |  | 1.018 | 1.002-1.034 | Lag 1 |
| O_3_ | Adjusted for CO | 1.006 | 0.989-1.023 | Lag 1 |
|  | Adjusted for NO_2_ | 1.009 | 0.992-1.026 | Lag 1 |
|  | Adjusted for PM_10_ | 1.01 | 0.993-1.027 | Lag 1 |
|  | Adjusted for PM_2.5_ | 1.009 | 0.993-1.026 | Lag 1 |
|  | Adjusted for SO_2_ | 1.01 | 0.993-1.027 | Lag 1 |
| PM_10_ |  | 1.013 | 1.004-1.022 | Lag 5 |
|  | Adjusted for O_3_ | 1.014 | 1.005-1.023 | Lag 5 |
|  | Adjusted for SO_2_ | 1.012 | 1.002-1.022 | Lag 5 |
| PM_2.5_ |  | 1.015 | 1.003-1.028 | Lag 5 |
|  | Adjusted for O3 | 1.016 | 1.003-1.028 | Lag 5 |
|  | Adjusted for SO2 | 1.012 | 0.999-1.026 | Lag 5 |
| SO_2_ |  | 1.13 | 1.041-1.227 | Lag 7 |
|  | Adjusted for CO | 1.076 | 0.982-1.178 | Lag 7 |
|  | Adjusted for NO_2_ | 1.08 | 0.987-1.182 | Lag 7 |
|  | Adjusted for O_3_ | 1.119 | 1.028-1.218 | Lag 7 |
|  | Adjusted for PM_10_ | 1.09 | 0.993-1.196 | Lag 7 |
|  | Adjusted for PM_2.5_ | 1.085 | 0.988-1.193 | Lag 7 |

**Supplementary Table 5.** RRs of hospitalization attributable to air pollution (temporal degrees of freedom: 5–9)

| Pollutant | Variable | **Temporal degrees of freedom** | | | | | | | | | |
| --- | --- | --- | --- | --- | --- | --- | --- | --- | --- | --- | --- |
|  |  | **5** | | **6** | | **7** | | **8** | | **9** | |
|  |  | **RR** | **95%CI** | **RR** | **95%CI** | **RR** | **95%CI** | **RR** | **95%CI** | **RR** | **95%CI** |
| CO | lag_0 | 0.99 | 0.9-1.089 | 0.988 | 0.897-1.088 | 0.991 | 0.9-1.09 | 0.991 | 0.899-1.091 | 0.988 | 0.896-1.09 |
|  | lag_1 | 1.313 | 1.123-1.535 | 1.317 | 1.127-1.539 | 1.312 | 1.121-1.535 | 1.285 | 1.091-1.515 | 1.276 | 1.081-1.506 |
|  | lag_2 | 1.221 | 1.025-1.453 | 1.23 | 1.035-1.462 | 1.228 | 1.032-1.462 | 1.203 | 1.005-1.44 | 1.191 | 0.993-1.429 |
|  | lag_3 | 1.306 | 1.104-1.545 | 1.309 | 1.108-1.546 | 1.308 | 1.106-1.546 | 1.291 | 1.086-1.534 | 1.279 | 1.072-1.525 |
|  | lag_4 | 1.24 | 1.048-1.467 | 1.24 | 1.049-1.465 | 1.239 | 1.048-1.466 | 1.227 | 1.031-1.459 | 1.219 | 1.022-1.455 |
|  | lag_5 | 1.221 | 1.032-1.446 | 1.219 | 1.03-1.444 | 1.219 | 1.029-1.444 | 1.207 | 1.014-1.436 | 1.202 | 1.009-1.432 |
|  | lag_6 | 1.284 | 1.09-1.513 | 1.278 | 1.083-1.507 | 1.276 | 1.08-1.507 | 1.231 | 1.019-1.487 | 1.23 | 1.02-1.484 |
|  | lag_7 | 1.339 | 1.129-1.588 | 1.336 | 1.127-1.584 | 1.327 | 1.116-1.577 | 1.263 | 1.046-1.525 | 1.259 | 1.043-1.519 |
|  |  |  |  |  |  |  |  |  |  |  |  |
| NO_2_ | lag_0 | 1.021 | 0.989-1.054 | 1.019 | 0.987-1.052 | 1.019 | 0.987-1.052 | 1.017 | 0.985-1.05 | 1.016 | 0.984-1.049 |
|  | lag_1 | 1.012 | 0.981-1.045 | 1.013 | 0.982-1.045 | 1.012 | 0.981-1.045 | 1.009 | 0.978-1.042 | 1.007 | 0.976-1.04 |
|  | lag_2 | 1.001 | 0.969-1.034 | 1.001 | 0.969-1.034 | 1.001 | 0.968-1.034 | 0.997 | 0.965-1.03 | 0.995 | 0.962-1.028 |
|  | lag_3 | 1 | 0.968-1.034 | 1.001 | 0.969-1.035 | 1 | 0.968-1.034 | 0.996 | 0.964-1.03 | 0.994 | 0.961-1.028 |
|  | lag_4 | 1.001 | 0.969-1.034 | 1.001 | 0.97-1.034 | 1.001 | 0.969-1.034 | 0.995 | 0.963-1.029 | 0.993 | 0.96-1.026 |
|  | lag_5 | 1.016 | 0.984-1.05 | 1.016 | 0.984-1.049 | 1.016 | 0.984-1.049 | 1.013 | 0.981-1.047 | 1.012 | 0.979-1.046 |
|  | lag_6 | 1.028 | 0.995-1.061 | 1.027 | 0.995-1.06 | 1.027 | 0.994-1.06 | 1.023 | 0.99-1.057 | 1.022 | 0.989-1.057 |
|  | lag_7 | 1.044 | 1.011-1.077 | 1.044 | 1.011-1.078 | 1.044 | 1.011-1.078 | 1.042 | 1.009-1.076 | 1.041 | 1.008-1.075 |
|  |  |  |  |  |  |  |  |  |  |  |  |
| O_3_ | lag_0 | 1.006 | 0.99-1.022 | 1.006 | 0.991-1.022 | 1.006 | 0.991-1.022 | 1.007 | 0.992-1.023 | 1.007 | 0.991-1.023 |
|  | lag_1 | 1.018 | 1.002-1.034 | 1.018 | 1.002-1.034 | 1.018 | 1.002-1.034 | 1.019 | 1.003-1.035 | 1.019 | 1.003-1.035 |
|  | lag_2 | 1.008 | 0.992-1.025 | 1.008 | 0.992-1.025 | 1.008 | 0.992-1.025 | 1.009 | 0.993-1.026 | 1.009 | 0.993-1.026 |
|  | lag_3 | 0.997 | 0.981-1.013 | 0.997 | 0.981-1.013 | 0.997 | 0.981-1.013 | 0.998 | 0.982-1.014 | 0.998 | 0.983-1.014 |
|  | lag_4 | 1.004 | 0.988-1.021 | 1.004 | 0.988-1.021 | 1.004 | 0.988-1.021 | 1.006 | 0.99-1.023 | 1.007 | 0.991-1.023 |
|  | lag_5 | 0.999 | 0.983-1.015 | 0.999 | 0.983-1.015 | 0.999 | 0.983-1.015 | 1 | 0.984-1.016 | 1.001 | 0.985-1.017 |
|  | lag_6 | 1.009 | 0.993-1.025 | 1.009 | 0.993-1.025 | 1.009 | 0.993-1.025 | 1.011 | 0.995-1.027 | 1.01 | 0.995-1.026 |
|  | lag_7 | 1.006 | 0.99-1.023 | 1.007 | 0.991-1.023 | 1.007 | 0.991-1.023 | 1.008 | 0.992-1.024 | 1.008 | 0.992-1.023 |
|  |  |  |  |  |  |  |  |  |  |  |  |
| PM_10_ | lag_0 | 1.005 | 0.996-1.014 | 1.004 | 0.995-1.013 | 1.004 | 0.995-1.013 | 1.003 | 0.994-1.013 | 1.003 | 0.994-1.012 |
|  | lag_1 | 1.004 | 0.995-1.013 | 1.004 | 0.995-1.013 | 1.004 | 0.995-1.013 | 1.003 | 0.994-1.012 | 1.002 | 0.993-1.011 |
|  | lag_2 | 1 | 0.991-1.009 | 1 | 0.991-1.01 | 1 | 0.991-1.01 | 0.999 | 0.99-1.009 | 0.999 | 0.989-1.008 |
|  | lag_3 | 1.005 | 0.996-1.015 | 1.006 | 0.997-1.015 | 1.006 | 0.996-1.015 | 1.004 | 0.995-1.014 | 1.004 | 0.994-1.013 |
|  | lag_4 | 1.009 | 1-1.018 | 1.009 | 1-1.018 | 1.009 | 1-1.018 | 1.008 | 0.999-1.017 | 1.007 | 0.998-1.017 |
|  | lag_5 | 1.013 | 1.004-1.022 | 1.013 | 1.004-1.022 | 1.013 | 1.004-1.022 | 1.012 | 1.003-1.021 | 1.009 | 1-1.019 |
|  | lag_6 | 1.01 | 1.001-1.019 | 1.01 | 1.001-1.019 | 1.01 | 1.001-1.019 | 1.008 | 0.998-1.018 | 1.008 | 0.998-1.018 |
|  | lag_7 | 1.011 | 1.002-1.02 | 1.01 | 1.001-1.02 | 1.01 | 1.001-1.019 | 1.008 | 0.998-1.017 | 1.007 | 0.998-1.017 |
|  |  |  |  |  |  |  |  |  |  |  |  |
| PM_2.5_ | lag_0 | 1.008 | 0.996-1.021 | 1.007 | 0.995-1.02 | 1.008 | 0.995-1.02 | 1.007 | 0.994-1.019 | 1.006 | 0.994-1.019 |
|  | lag_1 | 1.004 | 0.992-1.016 | 1.004 | 0.992-1.017 | 1.005 | 0.992-1.017 | 1.003 | 0.991-1.016 | 1.003 | 0.99-1.015 |
|  | lag_2 | 0.999 | 0.987-1.012 | 1 | 0.987-1.012 | 1 | 0.988-1.013 | 0.999 | 0.986-1.011 | 0.998 | 0.985-1.011 |
|  | lag_3 | 1.007 | 0.995-1.02 | 1.007 | 0.995-1.02 | 1.007 | 0.995-1.02 | 1.006 | 0.993-1.018 | 1.005 | 0.992-1.018 |
|  | lag_4 | 1.01 | 0.998-1.022 | 1.01 | 0.998-1.022 | 1.01 | 0.997-1.022 | 1.008 | 0.995-1.02 | 1.007 | 0.994-1.02 |
|  | lag_5 | 1.016 | 1.004-1.028 | 1.015 | 1.003-1.028 | 1.015 | 1.003-1.028 | 1.015 | 1.003-1.027 | 1.015 | 1.002-1.027 |
|  | lag_6 | 1.015 | 1.003-1.027 | 1.014 | 1.002-1.026 | 1.015 | 1.002-1.027 | 1.011 | 0.997-1.024 | 1.011 | 0.997-1.024 |
|  | lag_7 | 1.015 | 1.003-1.027 | 1.015 | 1.003-1.027 | 1.014 | 1.002-1.027 | 1.011 | 0.998-1.024 | 1.01 | 0.997-1.024 |
|  |  |  |  |  |  |  |  |  |  |  |  |
| SO_2_ | lag_0 | 1.062 | 0.979-1.151 | 1.057 | 0.974-1.146 | 1.057 | 0.975-1.146 | 1.053 | 0.971-1.142 | 1.051 | 0.968-1.14 |
|  | lag_1 | 1.091 | 1.007-1.182 | 1.093 | 1.009-1.184 | 1.092 | 1.008-1.183 | 1.084 | 1-1.175 | 1.08 | 0.996-1.17 |
|  | lag_2 | 1 | 0.92-1.087 | 1.002 | 0.922-1.089 | 1.001 | 0.921-1.087 | 0.994 | 0.914-1.08 | 0.991 | 0.911-1.077 |
|  | lag_3 | 1.015 | 0.932-1.106 | 1.018 | 0.934-1.108 | 1.015 | 0.932-1.106 | 1.004 | 0.921-1.095 | 0.999 | 0.916-1.09 |
|  | lag_4 | 1.034 | 0.949-1.127 | 1.035 | 0.95-1.127 | 1.033 | 0.948-1.125 | 1.018 | 0.933-1.112 | 1.013 | 0.927-1.106 |
|  | lag_5 | 1.072 | 0.985-1.167 | 1.071 | 0.984-1.165 | 1.07 | 0.983-1.165 | 1.062 | 0.975-1.156 | 1.058 | 0.97-1.153 |
|  | lag_6 | 1.106 | 1.019-1.2 | 1.101 | 1.014-1.196 | 1.1 | 1.013-1.194 | 1.08 | 0.992-1.176 | 1.08 | 0.992-1.176 |
|  | lag_7 | 1.134 | 1.045-1.231 | 1.133 | 1.044-1.23 | 1.13 | 1.041-1.227 | 1.113 | 1.023-1.211 | 1.111 | 1.021-1.208 |
